# Germline and Somatic Changes Associated with the Development of Inherited and De Novo Pediatric Acute Myeloid Leukemia

**DOI:** 10.3390/genes16070798

**Published:** 2025-07-01

**Authors:** Scott C. Smith, Lei Zhang

**Affiliations:** 1 Department of Pathology, SUNY Upstate Medical University, Syracuse, NY 13210, USA; 2 Department of Pathology, Children’s Mercy Hospital, Kansas City, MO 64108, USA; lzhang@cmh.edu

**Keywords:** pediatric AML, inherited AML, de novo AML, genetic abnormalities, cytogenetics, cytogenomics

## Abstract

Acute myeloid leukemia (AML) accounts for only about 15–20% of pediatric leukemia and an overall incidence of 1.4 cases per 200,000 children under the age of 15 years. The majority of pediatric AML occurs de novo, often as the result of somatic first hits in utero. A minority of pediatric AML occurs in response to a predisposition syndrome, such as a bone marrow failure syndrome, or other inherited mutations and copy number changes. While the overall survival of pediatric patients with AML is approximately 70%, survival at the individual level is dependent on the abnormality detected either through cytogenomic analyses or sequencing for mutations in responsible genes. Indeed, de novo infant AML carries a more sobering prognosis than that of pediatric AML. This review describes many of the common genomic abnormalities associated with pediatric AML and characterizes their detection from a laboratory assessment perspective. Pediatric AML is primarily a disease of gene rearrangements rather than of gene mutations, and, as such, clinical cytogenetics takes a primary role.

## 1. Introduction

Acute myeloid leukemia (AML) is a hematological malignancy with an incidence that typically increases with age. The differentiation of myeloid precursors becomes impaired, resulting in the proliferation of hematopoietic stem cells (HSCs) and their progenitors (HSCPs), thereby leading to the accumulation of blasts [1,2,3,4]. The overall incidence of AML in the pediatric population is highest for infants at 1.5 per 100,000 individuals. For those aged 1–4 years, the incidence is 0.9 per 100,000 individuals, and for those aged 5–9, it is 0.4 per 100,000 individuals [5]. Overall survival approximates 60–70% [6]. In contrast, adult AML occurs with an incidence of 16.2 per 100,000 individuals after age 65, the median age of diagnosis.

Pediatric AML is not simply an early-onset adult AML. The de novo mechanisms of leukemogenesis in infancy or childhood remain elusive despite numerous hypotheses being provided. Mechanistic hypotheses have included multiple in utero exposures and parental age at conception, among others. Yet such proposed mechanisms lack an explanation for recurrent genomic rearrangements found only in pediatric AML. As such, while comparable, little space will be focused on the mechanisms of pediatric AML leukemogenesis here, and appropriate attention will be paid to the genetic abnormalities that delineate pediatric AML. Characterizing such differences also requires a discussion of heredity, predisposition, and susceptibility. Indeed, germline predisposition versus somatic predisposition, via an early exposure or other acquired abnormality, may have differing consequences. Further, pediatric AML due to heritable disorders accounts for only approximately 10% of all cases. To that point, many germline AML predisposition syndromes have a median onset in adulthood and may not be biologically comparable to de novo pediatric AML.

This review aims to characterize many of the genetic abnormalities associated with the development of pediatric AML and the diagnostic approaches necessary to identify them. These include the chromosomally visible and cryptic translocations, the copy number variations, and nucleotide-level gene mutations. This review begins with a comparison between adult and pediatric AML, discusses the diagnostic approaches appropriate to the malignancy, and identifies predisposition syndromes that lead to AML, such as inherited bone marrow failure syndromes, hereditary myeloid malignancies, and Down syndrome. The remainder of this review focuses on the de novo forms of pediatric AML and highlights many cytogenetically alterations and their resultant fusions, as well as the primary mutation targets in the genome.

### Pediatric AML Versus Adult AML

Pediatric AML is primarily de novo and is rarely preceded by clonal evolution of preleukemic myeloproliferative disease. In contrast, this is the predominant progression in adult AML [7]. While the leukemogenesis of the adult and pediatric forms of the disease may harness common genomic players, there are distinct differences. Approximately two-thirds of pediatric AML is cytogenetically abnormal, indicating the presence of visually identifiable, and often recurrent, chromosomal rearrangements, such as *KMT2A* (11q23) rearrangements. The remaining minority may possess cryptic rearrangements, such as *NUP98::NSD1* gene fusions, or nucleotide-level mutations, such as in *NPM1*, *FLT3* internal tandem duplication (-ITD), or biallelic mutations in *CEBPA.* Indeed, there is an inverse relationship between age and the presence of visually identifiable translocations in all of AML. Translocations and non-normal karyotypes are found most in infants (up to age 2 years), followed by children up to age 14. They are least common in adults forty years and older [8]. The translocation t(8;21), which results in a *RUNX1::RUNX1T1* fusion and carries a favorable prognosis, is largely a pediatric rearrangement, found in 15% of pediatric AML but only 5% of adult AML. In contrast, somatic mutations in protooncogenes or tumor suppressor genes account for the majority of adult AML [9].

Since the accumulation of somatic mutations in hematological stem cells is proportional with age, AML initiating events are possibly passenger mutations that later acquire more potent lesions [10,11]. The childhood onset of AML suggests the involvement of predisposing mutations, yet apart from a group of germline predisposition syndromes, including the whole chromosome gain of 21 [12,13], the etiology of de novo pediatric AML remains unknown [14]. Even so, predisposition syndromes often have disease onsets outside of childhood. Indeed, myelodysplastic neoplasm (MDS)/acute leukemia (AL, including lymphoblastic leukemia and myeloid leukemia) may present in adulthood without a prior diagnosis of a bone marrow failure syndrome and without congenital anomalies.

While the majority of pediatric acute leukemia with very early onset age is of the lymphoblastic type, it has long been hypothesized that there is an in utero ‘first hit’ in the sequential two somatic hit hypothesis that, over time, develops into leukemia. In utero germline mutation first hits have been hypothesized to arise from exposures to paint, pesticides, and a farm animal environment [15,16,17,18], as well as advanced parental age [19], among others. In utero somatic first hits, the timing of a sequential second hit, like in numerous other autosomal dominant hereditary cancers, cannot be predicted, and, therefore, infant or childhood onset is not a requirement. Indeed, twin studies have revealed germline mutations resulting in concordant leukemias with onsets many years apart [20,21,22]. Further, there is an element of incomplete penetrance as some mutations manifesting in both twins were inherited from healthy parents without leukemia [20].

Aging has been hypothesized to impair HSCP differentiation resulting in adult AML. Features of aging have been studied in HSCs, such as genomic instability and the accumulation of unrepaired double-stranded breaks [23], reduced progenitor regenerative capacity [1,3], and lineage changes [24]. The accumulation of unrepaired DNA damage in aged HSCs is significant compared to cells derived from cord blood [23], thereby potentially paving the way to the accumulation of mutations and subsequently to hematopoietic dysfunction [10]. In contrast, infant HSCs are not quiescent like their adult relatives and, in humans, retain features of fetal hematopoiesis until three years of age [25,26,27]. For infant and pediatric AML, the mutational burden itself is abnormal [11]. Recent studies into the origin of pediatric AML suggest that some HSCPs harbor a greater mutation burden compared to normal HSCPs, owing to a high proliferation rate and increased oxidative stress. Such high mutation progenitors are comparably more committed to differentiate and, therefore, contribute to better overall outcomes [11].

## 2. Diagnostic Approaches

**Figure 1:**
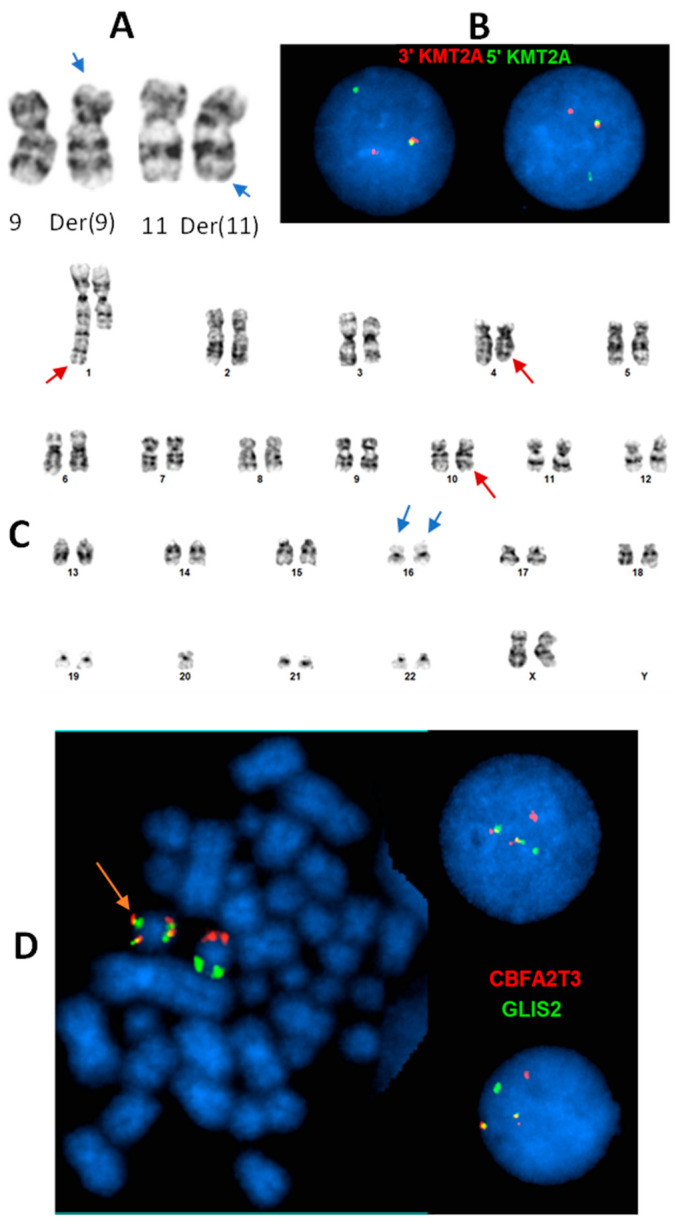
Common KMT2A (11q23.3) rearrangement and cryptic inv(16)(p13.3;q24.3)(CBFA2T3::GLIS2 fusion) in infant AML. (**A**): t(9;11)(p21.3;q23.3) identified by chromosome analysis in a 6-month female with AML. Arrows: subtle abnormal 9p and 11q. (**B**): Interphase FISH using *KMT2A* break-apart probe (green: 5″*KMT2A*; red: 3′*KMT2A*) confirms *KMT2A* rearrangement by the t(9;11). (**C**): An apparently normal pair of chromosome 16 homologs (blue arrows) in a cell with other non-subtype specific clonal abnormalities (red arrows) in a 21-month female with AML. (**D**): Dual color, dual fusion FISH with *CBFA2T3* (red)/*GLIS2* (green) probe set identified inv(16) on a metaphase cell (orange arrow) and *CBFA2T3::GLIS2* fusion on both metaphase and interphase cells (two fusion, one red, one green signal pattern), which is subtype-defining associated with poor prognosis, and cryptic (was not recognized by chromosome analysis).

**Figure 2:**
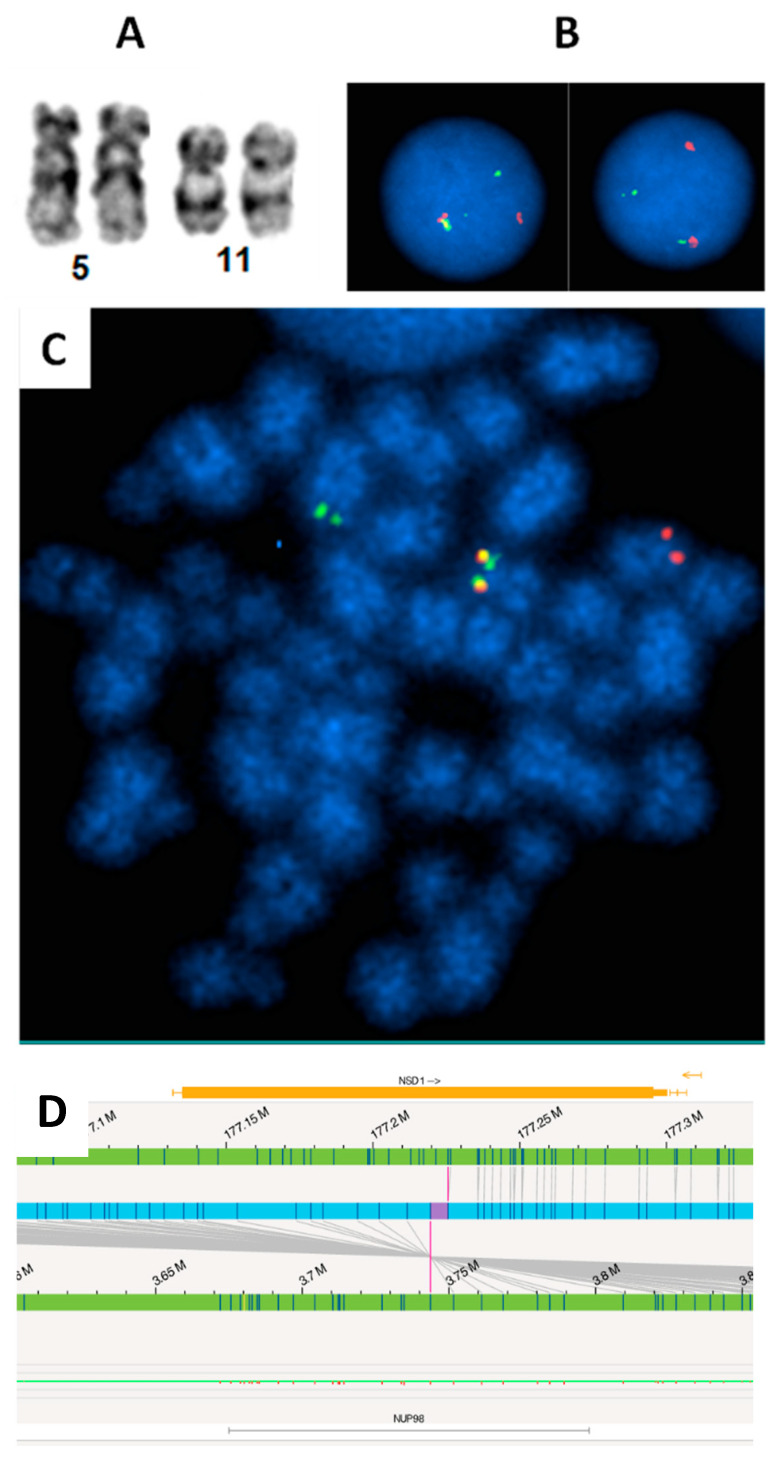
FISH revealed cryptic *NUP98* (11p15) rearrangement in a pediatric AML patient. (**A**): Apparently normal chromosome 11p in karyotype. (**B**): Interphase FISH using break-apart probe identified *NUP98* rearrangement (one fusion, one red, one green signal pattern). (**C**): Metaphase FISH suggests the *NUP98* rearrangement likely resulted from a t(5;11) with 3′*NUP98* probe translocated to the long arm of a chromosome, which is likely chromosome 5. (**D**). The *NUP98::NSD1* fusion was identified by Optical Genome Mapping (OGM). The upper and lower green tracks indicate the genomic locus of *NSD1* and *NUP98*, respectively. The blue track in the center indicates the fusion between the two genes, and the purple vertical lines spanning to the two green tracks show the fusion breaks on each green track.

Since the discovery of a relationship between the Philadelphia chromosome and chronic myelogenous leukemia (now chronic myeloid leukemia; CML) [28] and the subsequent identification of the chromosomal translocations, t(9;22) in CML [29], and t(8;21) in AML [30], clinical cytogenetics has been of paramount importance in hematological malignancies. As evidence of this importance, it now includes technologies beyond chromosome analysis that are rooted in molecular biology, such as fluorescent in situ hybridization (FISH) and chromosomal copy number microarray (CMA). The modernization of clinical cytogenetics provides welcome additional diagnostic and prognostic information in the oncology setting, wherein chromosome dynamics continues to dictate prognosis and treatment options [8,20,31,32,33]. In recent years, artificial intelligence has further removed numerous technologist-required steps in the karyotyping process by eliminating the digital cutting of a metaphase spread, to immediately provide the technologist a karyogram to approve following its digital image capture [34]. Break apart or dual color, dual-fusion FISH probes specific to a host of chromosomal rearrangements have given the cytogeneticist the opportunity to identify rearrangements or cryptic translocations (Figure 1 and Figure 2). Locus-specific FISH probes can identify copy number alterations and, at the same time, provide higher resolution at the interrogated locus. CMA provides a quasi-genome-wide view, comparable to chromosome analysis, with a resolution of approximately 10–20 kilobases (kb) rather than 3–5 megabases (Mb), as identified in chromosome analysis [35]. Finally, in recent years, the use of optical genome mapping, which images long stretches of labeled DNA molecules, and/or long read sequencing, can characterize genomic partner rearrangements with precision. The use of this technology is particularly promising in the pediatric AML realm and is becoming more commonplace in laboratories as a diagnostic companion to CMA, FISH, and G-banding [36].

Even in the rapidly evolving world of nucleotide-level sequencing, the importance of modern clinical cytogenetics cannot be overstated in pediatric AML. Most pediatric AML possesses chromosomal abnormalities, which may be copy number variations or genomic rearrangements that define an entity or provide a prognosis. For example, somatic or germline gains of chromosome 21, or somatic deletions of the long arm of chromosome 7 (7q deletion), are common recurrent copy number variations in pediatric AML. Examples of genomic rearrangements include the t(15;17) translocation, which creates a *PML::RARA* fusion transcript in acute promyelocytic leukemia (APL), and the rearrangement of the *KMT2A* gene located at 11q23. These may easily be recognizable in a karyotype by individuals trained in cytogenetics. Such abnormalities may be assisted in the speed of detection by the use of locus specific or dual-color, dual-fusion FISH probes, respectively. The 7q deletion is a prognostic marker, while the t(15;17) is diagnostic for APL and should be identified immediately in a patient due to the risk of disseminated intravascular coagulation (DIC). As such, rapid FISH should be prioritized in any patient where there is suspicion of APL or DIC. APL is treatable, with an excellent prognosis using all-trans retinoic acid (ATRA).

The modern molecular-based assays in a cytogenetics/cytogenomics laboratories often include CMA, which allows for the detection of copy number variants at a much higher resolution than either chromosome analysis or FISH. Further, CMA may also identify regions of homozygosity (ROH)/loss of heterozygosity (LOH). The detection of ROH/LOH can be especially useful if observed somatically. Copy-neutral LOH in the *TET2* locus, for example, may indicate the presence of a nucleotide-level mutation that has been endoreduplicated [37,38]. Such a finding would suggest that there is a biallelic *TET2* mutation. In such a case, sequencing the locus would be necessary to verify the finding. Given the copy-neutral nature of such a finding, only single-nucleotide polymorphism (SNP) arrays would be able to detect this abnormality. G-banding and FISH would both convey copy-neutral normal results. The primary shortcoming in CMA for pediatric AML, a disease predominantly about genomic rearrangements, is its inability to identify reciprocal translocations. CMA detects an absence or over-abundance of DNA, and in the latter case, does not have the capacity to determine where in the genome the abundance resides; an interstitial duplication of chromosome 21 could just as easily be tandem as it is located anywhere else in the genome. For such cases, the evaluation of a karyotype by G-banding or metaphase FISH is required. Fortunately, reciprocal rearrangements often carry small copy number variations that can be detected via an array. These can be a clue to the cytogeneticist that an intrachromosomal gene fusion or interchromosomal rearrangement is present. Indeed, the concept of ever-increasing resolution has spawned the popularity of optical genome mapping [36]. Another molecular-based assay in a cytogenetics/cytogenomics laboratory, which can overcome the shortcoming in CMA and has been implemented in an increasing number of laboratories, is optical genome mapping (OGM) technology [39]. By imaging ultra-long, single DNA molecules labeled with fluorescent dyes, OGM can not only detect copy number changes but also structural rearrangements, both unbalanced and balanced, including balanced translocations, inversions and insertions, at a high resolution. However, OGM has limitations in detecting smaller ROHs as well as structural rearrangements very close to the centromeres and telomeres [40].

Molecular testing has been incorporated into the AML diagnosis process after the recognition of frequent somatic mutations and their prognosis in AML patients with a normal karyotype [41]. The inclusion of next-generation sequencing (NGS) has led to a rapid expansion of genetic and epigenetic alterations associated with AML [42]. While NGS is becoming a test venue for new diagnoses of AML, other molecular tests, e.g., polymerase chain reaction (PCR), Sanger sequencing, multiplex ligation-dependent probe amplification (MLPA), and reverse transcriptase–polymerase chain reaction (RT-PCR), have been employed for confirmation, follow-up, or monitoring of genetic abnormalities in AML patients.

A comparison of the major testing technologies described above is summarized in Table 1. Each technology has its advantages in detecting genetic abnormalities contributing to diagnosis and prognosis stratification in AML. Although chromosome analysis plus a FISH panel analysis remains the major test venue in many laboratories, diagnostic approaches vary among different labs with different resources. Genetic testing should encompass all abnormalities that define the diagnosis and risk categories in AML according to the World Health Organization (WHO) classification and international consensus classification (ICC) [43,44]. The National Comprehensive Cancer Network (NCCN) guidelines and several review articles [45,46,47,48] may be referenced when considering a diagnostic algorithm.

## 3. Inherited Forms of Pediatric AML

Germline susceptibility to AML is defined by inherited bone marrow failure syndromes (IBMFSs) and hereditary myeloid malignancies (HMMs). The genes that define IBMFSs are often disease-causing in a recessive or X-linked manner, while those of HMMs are inherited in an autosomal-dominant manner (Table 2). This latter group of genes is comparable to other inherited cancers by their dominant inheritance pattern. Pathogenic constitutional (germline) variants identified by sequencing these genes may be identified in a clinical laboratory due to a variant allele frequency (VAF) that approaches 50%, if paired germline and somatic sequencing is not available [49]. Unlike IBMFS, patients predisposed to HMMs do not present with characteristic clinical features in childhood or adolescence. Another discerning feature is that each of the bone marrow failure syndromes has multiple causal genes defining a condition, while each HMM points to a single gene as the principal [13,50,51]. Indeed, the seven HMMs are mediated by mutations in one of seven genes (Table 2). Like the genes associated with IBMFS, the genes of the HMMs encode products with diverse functions, including hematopoietic transcription factors and leukemogenesis [50].

### 3.1. Inherited Bone Marrow Failure Syndromes

The group of life-threatening disorders known as IBMFSs usually presents in childhood with characteristic clinical features [13,50,51]. These disorders have a considerable risk for developing malignancies, including acute leukemias. Of these, Fanconi anemia is caused by deleterious mutations in an ever-growing number of genes responsible for genomic integrity and cell division, and it carries a very high risk of leukemia [52,53,54,55]. Other IBMFSs with a substantial likelihood of resulting in AML include dyskeratosis congenita and severe congenital neutropenia. Dyskeratosis congenita is mediated by 16 genes, primarily associated with telomere maintenance and ribosomal deficits, while there are seven genes responsible for severe congenital neutropenia [13,51].

### 3.2. Fanconi Anemia

Fanconi anemia (FA), the most common bone marrow failure predisposition syndrome, yields the highest increased risk of MDS/AL. Of FA patients, the prevalence of AML compared to other cases of leukemia was a striking 84% in one systematic review in the literature published from 1927 to 2012 [53]. Yet, one-quarter of patients do not present with characteristic features, such as short stature, café au lait spots, hyper- and hypopigmentation, microcephaly, microphthalmia, and abnormal thumbs, with or without hypoplastic radii [12,56,57]. For patients lacking the common features, pancytopenia may be the only alerting symptom. Moreover, there may be no other clinical feature of FA in these patients until their fifth decade of life [53].

The diagnosis of FA can be achieved using conventional cytogenetics or modern molecular techniques. FA may be diagnosed by observing increased chromosome breakage and radial forms on the cytogenetic testing of lymphocytes following exposure to crosslinking agents, such as diepoxybutane (DEB) and mitomycin C (MMC) [52,53,57]. Molecular tools are likely more commonly employed to diagnose FA, given that all BMF syndromes could be assessed at once if necessary. Modern molecular methods can be greatly beneficial in diagnosing hereditary disease. Next-generation sequencing may be used to identify copy number or nucleotide-level biallelic deleterious variants in any of the genes associated with the autosomal recessive form of the disorder. Twenty-one genes are associated with the autosomal-recessive forms of FA. Single deleterious variants in *RAD51* may be diagnostic in the autosomal-dominant form of the disorder. A *FANCB* hemizygous deleterious variant would be expected to cause the X-linked recessive form of FA [52].

The protein products of the 23 FA genes are among the proteins tasked with genome integrity. It, therefore, stands to reason that deleterious variants in any of the FA genes convey an exceedingly high increased risk of all cancers, not just MDS/AL. Of the genes most associated with FA, three (*FANCA*, *FANCC*, *FANCG*) account for an estimated 82% of all FA, and the product of each of these is associated with the FA core complex assembly [52]. Assembly of the multiprotein FA core complex is mediated by the activation and subsequent signaling of ATR [54] and CHK1 [58] in the DNA damage response. Recurrent acquired chromosome abnormalities in FA include gains of 1q and 3q due to unbalanced translocations and cryptic 21q translocations involving *RUNX1* [59,60].

### 3.3. Hereditary Myeloid Malignancies

Compared to the general population, a diagnosis of an HMM typically has an early onset, yet most are not diagnosed until adulthood. Approximately 10% of individuals with hematological malignancies possess a germline susceptibility mutation [13,61]. Table 2 identifies the seven genes associated with hereditary myeloid malignancies, the associated malignancy, and potential congenital clinical features. Of these, the mutations in *CEBPA* manifest AML without progression from MDS, separating it from the otherwise difficult-to-segregate phenotypic features observed from mutations in all other associated genes.

### 3.4. Familial AML with Mutated CEBPA

Familial AML with mutated *CEBPA* (FAMC) is unlike most other HMMs in that it is an AML. The other HMMs are not limited to AML but may also include myelodysplastic and myeloproliferative neoplasms, as well as lymphoblastic leukemia [12,13]. Up to 15% of pediatric and adult AML is mediated by mutations of *CEBPA.* Germline *CEBPA* mutations may develop into early-onset AML following subsequent somatic mutation acquisition [62,63]. Still, FAMC has a variable age of onset, with reports of patients as young as 2 years old and as old as 46 years [12,63,64]. Germline N-terminus mutations have been observed with nearly complete penetrance and a median age of onset of 25 years, with the range beginning as early as infancy (under 2 years) [65].

### 3.5. Down Syndrome

The most common inherited condition predisposing an infant to AML is Down syndrome. Down syndrome (DS) is a common genetic condition with an incidence of 1 in 600 to 1 in 800 live births, caused by an extra copy of chromosome 21 (trisomy 21). While the development of acute leukemia is dramatically increased in all individuals with DS, the increased risk of developing AML in children with DS is 150-fold that of an individual without DS. Compared to an up to 20-fold increased risk of developing acute lymphoblastic leukemia/lymphoma (ALL), AML is the predominate form of leukemia in children with DS younger than 4 years of age [66].

The congenital anomalies and risks associated with DS result from the extra whole copy of chromosome 21. One consequence of this aneuploidy is the increased expression of genes located on chromosome 21, including some associated with hematopoietic development, such as *RUNX1*; however, increased expression does not provide a full accounting of the consequences. Approximately 5% of newborns with DS develop transient abnormal myelopoiesis (TAM) from an acquired *GATA1* truncating mutation that results in a short isoform, GATA1s, that lacks the N-terminal transactivation domain [67,68,69,70,71]. The acquired mutation is associated with trisomy 21; however, *GATA1* itself resides at Xp11.23. Approximately 25% of neonates with DS possess *GATA1s* [72], and somatic mutations resulting in *GATA1s* have been observed in acute megakaryoblastic leukemia (AMKL) in children without DS but with an acquired trisomy 21 [73,74,75]. TAM precedes AML in DS and can often undergo spontaneous remission.

There are also consequences of aneuploidy outside the realm of hematopoietic development. Copper–zinc superoxide dismutase (*SOD1*) resides on chromosome 21 and is, therefore, overexpressed in Down syndrome. SOD1 catalyzes the conversion of superoxide into hydrogen peroxide and oxygen. Hydrogen peroxide is subsequently converted to water by glutathione peroxidase and catalase. The overexpression of *SOD1* without concomitant increases in catalase and glutathione peroxidase allows peroxides to accumulate and leads to the production of hydroxyl radicals [76,77,78,79,80]. Increased SOD1 activity has been linked to oxidative damage and apoptotic increases [76,78,79,81]. Similarly, the increased sensitivity to cytosine arabinoside (ara-C) and daunorubicin from ara-C triphosphate in DS myeloblasts has been identified to be in response to the overexpression of cystathionine-beta-synthase (CBS), due to its gene residing on chromosome 21. CBS overexpression favors ara-C conversion to ara-C triphosphate (ara-CTP), thereby increasing the nucleoside analog’s incorporation into DNA [82]. The result is the extreme sensitivity to leukemic treatment in patients with DS, especially to cytarabine, etoposide, and anthracyclines [66,71,83]. Interestingly, a recent study identified reduced genomic integrity in the hematopoietic progenitors of individuals with DS. Copy number gains and losses without clonal expansion were revealed as contributing factors in the mechanisms leading to AML in DS [84].

## 4. De Novo Pediatric AML

The majority of pediatric AML develops de novo. Unlike AML in adults, which typically follows from myelodysplastic syndrome or genotoxic exposure, the clonal evolution of preleukemic myeloproliferative diseases is rare in the pediatric population [5,32]. Generally, infant AML is characterized as a diagnosis up to two years of age. This definition is based on evidence that there are similar disease patterns in clinical characteristics and genetic abnormalities among this age group in the disease population [85,86]. In addition, there is a high prevalence of unfavorable risk factors and therapy-related toxicity in infant AML compared to older pediatric patients. Indeed, infant AML results in a higher proportion of cases with acute megakaryoblastic leukemia, and some genomic rearrangements are exclusive to the infant population [5,32,85,86,87,88,89]. While some genomic abnormalities are shared among those younger than age two years and those older, the proportions often differ. Unfortunately, it is rare that large cohort studies characterize age groups by infancy (0–2) and pediatric (>2). It is more common to observe AMKL set aside as a category, perhaps presuming infant AML to predominate in that category. Drilling down by age and AML subtype remains ideal, yet, by following that method, some cohorts will lack the statistical power required.

One recent study with age information in the supplemental information provides evidence of frequency differences of structural rearrangements [90]. The authors characterized structural rearrangements in ages 0–3, >3–10, and >10 years using RNA-seq data. *KMT2A* rearrangements were more prevalent in infant myeloblastic or monocytic AML compared to older patients, whereas *CBFB::MYH11* fusions were slightly more prevalent in older patients. *RUNX1::RUNX1T1* fusions were almost non-existent in the infant group compared to the older groups. Indeed, of 887 patients ranging in age from 0 to 23.5 years, fusions or structural variants were observed in nearly 71% of patients, with *RUNX1::RUNX1T1*, *CBFB::MYH11*, and *NUP98* rearrangements predominating following all *KMT2A* rearrangements [90]. Another recent cohort study that employed RNA-seq technology to identify driver alterations also supports historical data showing that *KMT2A* rearrangements remain the most prevalent across all pediatric age groups [90,91,92]. Only *PML::RARA* outperformed *NUP98* rearrangements in frequency among all age groups [92].

### 4.1. Infant AML

Infant AML is a distinct entity that occurs at a frequency of up to 25% of all pediatric AML. The predominant form in infants is AMKL, which is rare among other non-DS age groups [14,87]. AMKL is characterized by a blast count equal to 20% or greater with megakaryocytic differentiation [93]. Classifying AMKL by genetic abnormality is prognostic, and inferior outcomes are often identified in cohort studies. Indeed, many de novo cases can now be classified by their genomic rearrangements, t(1;22)(p13;q13) (*RBM15::MRTFA*), *KMT2A* (11q23) rearrangements (Figure 1A,B), inv(16)(p13.3q24.3) (*BFA2T3::GLIS2*) (Figure 1C,D), and t(5;11)(q35;p15) (*NUP98::KMD5A*) (Table 3). Prior to more advanced molecular techniques becoming mainstream in genetics laboratories, rearrangements involving the *KMT2A* locus or the t(1;22) translocation were the most commonly identified abnormalities in non-DS pediatric AMKL [94,95,96,97]. At that time, most karyotypes were normal and, therefore, were likely expected to result from nucleotide-level mutations. Instead, many recurrent gene fusions were later discovered and found to be cytogenetically cryptic. Now, such rearrangements can be identified using dual-color, dual-fusion FISH probes or break-apart FISH probes. Cohorts, therefore, differ in the frequency of rearrangements most commonly occurring that are associated with AMKL. Indeed, *KMT2A* rearrangements, *NUP98* fusions, *RBM15:MRTFA* fusions, and *CBFA2T3::GLIS2* fusions are the most common [14,93,94,98,99]. Other methods in recent years have become increasingly popular to identify the cryptic fusions (*CBFA2T3::GLIS2*, *NUP98::KMD5A*, *KMT2A::MLLT10*). These may include optical genome mapping, long-read sequencing, RNA sequencing, and, under the correct conditions, chromosomal microarray.

RNA-sequencing in recent cohort studies has resulted in new molecular categories. These general molecular categories are typically associated with cryptic fusions that result in aberrant gene expression. Two such categories, *GLIS* and *HOX* rearrangements, are almost entirely absent in patients aged three years and older [90]. Both the *GLIS* and *HOX* rearrangements were determined to fall into high-risk groups. The *HOX* rearrangements could co-occur with *KMT2A*, *KAT6A*, *NUP98* rearrangements, and *DEK::NUP214* fusions, as well as *FLT3*, *WT1*, *KRAS*, and *NRAS* mutations. *CBFAT23::GLIS2* is the most frequently occurring *GLIS2* rearrangement in AMKL and was found to be mutually exclusive to *UBTF* tandem duplications, *FUS::ERG* fusions, *CBFB* insertions, and others [90,93].

Although there are reports of additional cytogenetic abnormalities being observed in conjunction with recurrent AMKL-causing genomic rearrangements, specific correlations, such as copy number alterations and the like, have not been well established [89,98]. The benefit of such correlations could not be understated, as a cytogeneticist may be alerted to one of the cryptic rearrangements if an otherwise-normal karyotype revealed an associated copy number abnormality. Such associations have been observed in abnormalities of both the myeloid and lymphoid lineages, such as +22 with inv(16)/t(16;16) [*CBFB::MYH11*] in the former. While not every case would be expected to also possess another abnormality that could be identified chromosomally, an association with a cryptic or difficult-to-identify abnormality may trigger further targeted evaluation. Indeed, a recent Children’s Oncology Group (COG) study repeatedly identified trisomy 3 and a paucity of complex karyotypes with the cryptic inversion, inv(16)(p13.3q24.3) [*CBFA2T3::GLIS2*] [93]. Gains of chromosome 21 are observed in approximately 10% of de novo pediatric AMKL cases, known as DS-like AMKL. Interestingly, such cases also possess *GATA1s* mutations, indicating their close connection to Down syndrome. A karyotype showing +21 would necessitate a constitutional evaluation of the infant to be reassured that the gain of chromosome 21 is limited to the blast cell population and, therefore, an acquired finding.

*KMT2A* (11q23) rearrangements are among the most common abnormality in pediatric AML and account for about 7% of infant AMKL (Table 3) [89,93,98]. With the known promiscuity associated with the *KMT2A* gene, shown to partner with greater than 80 other genes, rearrangements involving this gene are well documented in acute lymphoblastic and acute myeloid leukemias. Indeed, the universality of this gene being rearranged was addressed in its original name, *MLL* (mixed-lineage leukemia) [95]. The prominent *KMT2A* rearrangement partner, *MLLT3*, is the most common of all *KMT2A* rearrangements in infant AML and is associated with both myelomonocytic leukemia (AMML) and AMKL [87,94]. Other common *KMT2A* rearrangement partners in AMKL include *MLLT10*, *MLLT1*, and *MLLT6*, listed in order from most common to least, as identified in an AMKL cohort study published in 2016 [100].

Some genomic rearrangements heavily predominate or are even restricted to the infant population. The t(8;16)(p11;p13) translocation fuses *MYST3* with *CREBBP* and can be observed in a majority of infants and 28% of newborns [101]. Interestingly, Coenen et al. observed spontaneous remission in a subset of the newborn population with t(8;16) translocations [101]. An important rearrangement involving *ETV6* (12p13) is the t(7;12)(q36;p13) [*MNX1::ETV6*] translocation. It is restricted to the infant AML population and carries a poor prognosis. Like so many translocations in infant AML, this is cryptic and, therefore, is best evaluated using FISH or sequencing (RNA sequencing, long-read sequencing, optical genome mapping). Interestingly, the fusion transcript is detected in approximately 50% of cases, likely due to the heterogeneity of the 7q36 locus breakpoints that can even occur proximal to *MXN1* [102].

Genomic abnormalities associated with infant AMKL also include nucleotide-level mutations. ERG expression dysregulation is the mechanism of action for *CBFA2T3::GLIS2* fusion through essential target genes, such as *KIT*, which is controlled by both ERG and CBFA2T3::GLIS2 fusion [103]. Indeed, in a cohort of 44 pediatric non-DS de novo AMKL, 7% of the cohort possessed *KIT* mutations. Of the entire cohort, 16% had normal cytogenetics, and 38% carried gene mutations. Another 7% were found with *NRAS* mutations, 5% with *WT1* mutations, 2% with *KRAS* mutations. *FLT3* internal tandem duplications (-ITDs) were the most common finding (9%) after *GATA1* mutations (11%) [98]. Similarly, another cohort found RAS-related mutations were common with *KMT2A* rearrangements [8].

### 4.2. Pediatric De Novo AML

The age threshold of two years partitions infant AML from pediatric AML and, with it, many genetic differences. While *KMT2A* rearrangements continue to predominate in approximately 20% of cases, the majority of other cases are found to carry a t(15;17)(q24;q21) [*PML::RARA*] fusion, a t(8;21)(q22;q22) [*RUNX1::RUNX1T1*] fusion, or a *CBFB::MYH11* fusion, found in either inversion 16 [inv(16)(p13q22)] or translocation t(16;16)(p13;122). The sum prevalence of those rearrangements plus all *KMT2A* rearrangements, the most common of which is still the t(9;11) [*KMT2A::MLLT3*] fusion, accounts for 39–55% of pediatric AML [32]. Apart from the *KMT2A* rearrangements, all of these rearrangements carry a favorable prognosis. While the t(9;11) translocation involving *KMT2A* is often generalized as the most favorable of the *KMT2A* rearrangements and, therefore, carries an intermediate prognosis, three others are also intermediate risk. The t(1;11)(q21;q23) [*KMT2A::MLLT11*], t(11;19)(q23;p13.1) [*KMT2A::ELL*], and t(X;11)(q24;q23.3) rearrangements account for approximately 2–3% of pediatric AML [8,104,105,106]. Of those, only the t(9;11) rearrangement has a median age of onset in the infant period.

Other fairly common abnormalities include rearrangements of *NUP98* and abnormalities associated with 12p13, which include deletions of the 12p locus, fusions of *KMD5A*, and abnormalities of *ETV6*. Together, this group of abnormalities accounts for another 7–9% of pediatric AML [32,33,98]. The *NUP98* (11p15) rearrangements are rare, but the most common fusion partners are *NSD1* (5q35), resulting in a cryptic t(5;11)(q35;p15) translocation (Figure 2A,C), and *KDM5A* (12p13.3), resulting in a t(11;12)(p15;p13.3) quasi-cryptic translocation. The t(5;11) is often associated with *FLT3* internal tandem duplication and/or *WT1* mutations (Figure 2C). The t(11;12) is usually associated with AMKL and is observed approximately equally in both pediatric and infant AML [32,33].

Non-cryptic karyotypes with abnormalities commonly observed in myelodysplastic syndrome, such as monosomy 5/5q deletion, monosomy 7/7q deletion, trisomy 8, and complex karyotypes with greater than 3 chromosomal abnormalities are also present in pediatric AML. While abnormalities of chromosomes 5 and 7 are rare, accounting for only about 1–3% of all cases, acquired trisomy 8 is more common, with frequencies exceeding 10% [107]. The abnormalities of 5 and 7 are associated with adverse outcomes, while the prognosis of whole chromosome gains of 8 continues to be more nuanced, with varying results [32]. The identification of a +8 karyotype suggests that the hunt for a primary abnormality should continue, as it is typically a secondary change. Notably, complex karyotypes do not carry the same adverse prognosis in pediatric AML as has been established in adult AML, and they are typically associated with guarded outcomes [32].

Normal karyotypes in pediatric AML represent either cryptic translocations or, less commonly, nucleotide-level mutations within a selection of genes. Diagnostic approaches following a normal karyotype should involve a method that includes break-apart FISH probes for commonly rearranged genes, sequencing panels, and/or optical genome mapping/long-read sequencing. Similarly, allele-specific molecular assays may be employed for specific mutations or nucleotide-level indels or duplications. Indeed, *FLT3-ITD* has a frequency of approximately 10% in pediatric AML and is associated with unfavorable outcomes [31].

Sequencing panels typically include the most common targets of mutations, and, as such, a large net may be cast. Mutations in *TET2*, *NPM1*, *CEBPA*, *NF1*, *WT1*, *KIT*, *NRAS*, *KRAS*, *PTPN11* have been observed in pediatric AML [37,86,108,109]. *NPM1* and *CEBPA* mutations are usually associated with favorable outcomes and more common in patients aged three years and older. *CEBPA* biallelic mutations, *PTPN11*, and *NRAS* mutations are found in approximately equal frequencies between infant and pediatric AML patients, while *KRAS* mutations predominate in the latter [86].

Biallelic *CEBPA* mutations, occurring in the N-terminal transcription activation domains and in the C-terminal basic leucine zipper region, are leukemogenic events in AML. Constitutional, first hit events have bene shown to occur at the N-terminal domain, to predispose individuals to developing AML [110]. A recent transcriptome study of 2958 patients aged 0–29.9 years enrolled in COG trials compared *CEBPA* mutation status to wildtype *CEBPA* and evaluated outcomes between the groups. C-terminal mutations and biallelic mutations were similar in the event-free (64%, 64%, respectively) and overall survival rates (89%, 81%, respectively) compared to non-mutated *CEBPA* [111]. That study further concluded that the presence of a C-terminal *CEBPA* basic leucine zipper mutation, regardless of biallelic or monoallelic mutation presence, carries a favorable status.

Deletions of target genes have also been identified in pediatric AML [108]. These deletions may be at the kilobase level, involving single exons, spanning multiple exons, or even targeting the entire gene. While tiny in comparison to the multi-megabase-level copy number alterations identified in karyotypes, these are large with respect to nucleotide-level deletions, duplications, or insertions identified via sequencing technologies. Sequencing panels not evaluating copy number alterations are not likely to detect large kilobase-level deletions, and, as such, these are much more assessable by CMA [35,108].

The diagnostic approach in de novo pediatric AML is best conducted in an algorithmic manner that plays the odds. Karyotype and FISH are typically the first approach and then, depending on the outcome, CMA. First-tier FISH should include translocations such as t(8;21), t(15;17), t(1;22), inv(16), *KMT2A* rearrangements, and copy number alterations (chromosomes 5/5q, 7/7q, 8). FISH would also be useful if the defined AML differentiation suggests that a cryptic translocation is likely (e.g., AMKL), in which case targeted probe sets may be employed. CMA can be helpful in identifying a loss of heterozygosity, especially involving recurrent target genes, intragenic and exonic deletions, and for kilobase-level copy number alterations that may be difficult to assess using sequencing techniques. Molecular approaches can be helpful if normal karyotypes are identified. Specifically, if FISH panels are limited, RNA sequencing or allele-specific assays may be helpful in identifying the numerous cryptic rearrangements, while DNA sequencing will be helpful in identifying gene mutations in the associated recurrent target genes.

## 5. Conclusions

Pediatric AML is a heterogenous disease, as defined by its differentiation, clinical presentation, and genetic abnormalities. Identifying the specific genetic abnormalities is of the utmost importance for diagnosis, prognosis, and treatment options. The technologies used have grown and developed over time and, with them, the identification of novel abnormalities. Karyotypically cryptic translocations can be assessed with older molecular techniques such as FISH or CMA. New molecular techniques are becoming more readily available, such that most clinical genetics laboratories now possess some level of sequencing capacity. Yet, most cases are still represented by a limited number of genomic rearrangements or gene targets and can be revealed using traditional cytogenetics methods. This review characterized many of the biomarkers with respect to differentiation, age of presentation, germline predisposition, and diagnostic technology.

**Figure 3:**
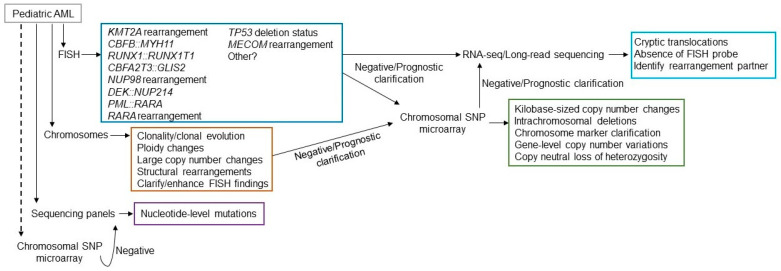
Possible testing algorithm for presentation of a pediatric AML patient. FISH, chromosome analysis, sequencing panels should all be considered as first tier testing (solid lines from patient), while chromosomal microarray may be considered first tier or for further clarification (dotted lines from patient). Available FISH probes may differ from laboratory to laboratory, but likely will include most of the probe sets listed. Many fusions are cryptic by chromosome analysis. The single-cell based technology, chromosome analysis, will provide evidence of clonality and clearly show clonal evolution. It will also provide megabase (Mb)-level copy number variation, changes in ploidy, and structural rearrangements that may be different than employed with FISH. Sequencing panels will include the genes most commonly mutated. Microarray will assist in identifying smaller copy number variations that chromosome analysis missed, clarify marker chromosomes, if identified, and show prognostic gene deletions. Plus it can detect LOH. If no structural rearrangements are observed and driver mutations by sequencing are not identified, RNA-sequencing, long-read sequencing, optical genome mapping, or comparable technologies may be beneficial if available.

The identification of recurrent abnormalities that define a pediatric AML entity necessitates a defined diagnostic approach. While the recurrent genomic abnormality diagnostic approach in predisposition syndromes is primarily focused on assessing the genes associated with a clinical phenotype, if one exists, the approach for de novo AML begins with the age of presentation and a karyotype. From there, it is a matter of employing the technologies available to characterize the entity, thereby providing diagnostic and/or prognostic information. Given that a majority of de novo pediatric AML has recurrent translocations and specific gene rearrangements, the diagnostic technologies in a typical clinical cytogenomics laboratory will largely be sufficient (Figure 3).

In a minority of pediatric AML, cryptic chromosomal translocations and gene mutations are the leukemogenic drivers, which may require recently adopted or developing technologies. While FISH can detect many cryptic fusions, it would be impossible to hybridize every possible dual-color/dual-fusion probe set to a patient specimen. When results are negative from other testing modalities, it may be valuable to obtain results from long-read sequencing, transcriptome sequencing, OGM, or other comparable technology may identify fusion partners. While this technology is currently available in a handful of laboratories, it gains more traction each year, as more and more laboratories share their success stories with its employment.

Recent cohort studies have highlighted mutually exclusive genomic aberrations in pediatric AML, which can be extremely helpful for the diagnostician. Although diagnostic exclusivity is part of the current algorithmic approach, one can imagine an approach that is much more precise and targeted using new, emerging data about AML. It is now up to laboratories to use this information to benefit patients. In the near future, chromosome analysis, DNA sequencing panels, FISH panels, and long-read technologies may be combined into testing algorithms that capitalize on mutual exclusivity to reach a diagnosis or prognosis more quickly and with fewer if/then steps.

**Table 2:** The genes associated with the autosomal dominant hereditary myeloid malignancies.

Gene	Syndrome	Age of Onset	Associated Malignancy	Associated Congenital Anomalies
*CEBPA*	Familial AML with mutated *CEBPA*	As young as 1.8 [21]	AML	None reported
*DDX41*	Familial AML with mutated *DDX41*	Mid to late adulthood [112]	MDS/AML, chronic myelomonocytic leukemia (CMML)	None reported
*RUNX1*	Familial platelet disorder with propensity to myeloid malignancies	Early childhood to late adulthood [113]	MDS/AML/T-cell acute lymphoblastic leukemia (T-ALL)	Thrombocytopenia, bleeding propensity
*ANKRD26*	Thrombocytopenia 2	Childhood to early adulthood [114]	MDS/AML	Thrombocytopenia, bleeding propensity
*ETV6*	Thromboycotpenia 5	Between 2 and 82 with average age of 22 and median age of 11 [115]	MDS/AML, CMML, B-cell ALL, plasma cell neoplasm	Aplastic anemia
*GATA2*	Familial MDS/AML with mutated GATA2	Early teens to early twenties [116]	MDS/AML/CMML	Neutropenia, monocytopenia, Emberger syndrome, MonoMAC syndrome
*SRP72*	Familial aplastic anemia with *SRP72*	Adolescent to later adulthood [117]	MDS	Aplastic anemia

**Table 3:** Recurrent findings in de novo infant AML.

Rearrangement	Gene Partners (If Known)	Defined Differentiation	Prognosis	Cytogenetic Detection
t(1;22)(p13.3;q13.1)	*RBM15::MRTFA*	AMKL	Poor	Cytogenetically visible
inv(16)(p13.3q24.3)	*CBFA2T3::GLIS2*	AMKL	Poor	Cryptic
t(5;11)(q35;p15)	*NUP98::KMD5A*	AMKL	Poor	Cryptic
t(9;11)(p21.3;q23.3)	*KMT2A::MLLT3*	AMKL/AMML	Intermediate	*KMT2A* break apart FISH probe
t(10:11)(p12.31;q23.3)	*KMT2A::MLLT10*	AMKL	Poor	*KMT2A* break apart FISH probe
t(11;17)(q23.3;q12–21)	*KMT2A::MLLT6*	AMKL/AMML	Poor	*KMT2A* break apart FISH probe

## Figures and Tables

**Table 1:** Comparison of major genetic technologies used for acute myeloid leukemia (AML) diagnostics in cytogenetics laboratories.

Technologies	Karyotyping	Fluorescent In Situ Hybridization (FISH)	Chromosomal SNP Microarray Array (CMA)	Optical Genome Mapping (OGM)
**Coverage**	Genome-wide	Targeted	Genome-wide	Genome-wide
**Specimen-Type**	Viable Cells	Viable or Fixed Cells	Viable or Fixed Cells	Viable Cells *
**Analysis Type**	Single Cell	Single Cell	Bulk Specimen	Bulk Specimen
**Resolution**	≥5~10 Mb	~70 kb–1 Mb	≥5 kb~200 kb	≥~500 bp~5 kb
**Sensitivity**	~10%	~2–5%	~10–15%	~10–15%
**CNV**	+	+	+	+
**Balanced SV**	+	+	-	+
**Unbalanced SV**	+	+	+	+
**CN-LOH**	-	-	+	Limited
**Triploidy**	+	+	+ (CMA)	+ (in VIA)
**Tetraploidy**	+	+	-	-

*: Viable cells can be frozen; CNV: copy number variant; SV: structural variant; CN-LOH: copy neutral loss of heterozygosity; SNP: single nucleotide polymorphism; VIA: VIA software (version number VIA), developed by BioDiscovery (San Diego, CA, USA).
